# Impact of ConcanavalinA affinity in the intracellular fate of Protein Corona on Glucosamine Au nanoparticles

**DOI:** 10.1038/s41598-018-27418-w

**Published:** 2018-06-13

**Authors:** Desirè Di Silvio, Alessandro Silvestri, Luigi Lay, Laura Polito, Sergio E. Moya

**Affiliations:** 1 Soft Matter Nanotechnology Group, CIC biomaGUNE, Paseo Miramon, 182, 20014 San Sebastian, Spain; 2 CNR – ISTM, Nanotechnology Lab., Via G. Fantoli 16/15, Milan, Italy; 3 Department of Chemistry, University of Milan, Via C. Golgi 19, Milan, Italy; 4 CRC Materiali Polimerici (LaMPo), University of Milan, Via C. Golgi 19, 20133 Milan, Italy; 5 CNR – ISTM, Nanotechnology Lab., Via G. Fantoli 16/15, 20138 Milan, Italy; 6 Present Address: Max-Planck Institute of Colloids and Interfaces, Potsdam-Golm, 14476 Germany

## Abstract

Biological fate and toxicity of nanoparticles (NPs) are connected to the interaction between NPs and the protein corona (PC) spontaneously forming around NPs in biological matrixes. PC is a dynamic entity that confers biological identity to NPs. In this work, fluorescence cross-correlation spectroscopy (FCCS) is used to study the impact of specific interactions between the NP surface and proteins on the intracellular fate of PC. The stability of the PC formed around glucosamide-functionalized Au-NPs from ConcanavalinA (ConA) or Bovine Serum Albumin (BSA) is characterized by FCCS. The NPs show higher affinity for ConA and competitive assays show that ConA easily exchanges BSA. A549 cells are exposed to glucosamide-functionalized Au-NPs with preformed ConA and BSA PCs. Intracellularly the frequency of cross-correlation for Au NPs with ConA PC remains constant to a 70% value until 24 h while for BSA it decreases to a 15% during the same period. FCCS measurements in several locations in the cell point out a different level of aggregation for the NPs with either ConA or BSA PCs. Our results show that the affinity of NPs functionalized with a ligand with affinity for a specific protein in bulk is retained intracellularly influencing NP fate and translocation.

## Introduction

The surface chemistry of nanoparticles (NPs) plays a fundamental role on their interaction with biomolecules and their fate in biological matrixes. The engineering of the NP surface is the usual strategy followed to control cellular uptake and trafficking of NPs and to confer the NPs with specific recognition properties^1–3^. A key aspect in the interaction of NPs with biological fluids and their biological fate is the formation of the so-called protein corona (PC)^4^. The corona results from the association of proteins to the NPs, forming a coating that shields the NP surface. The PC composition, the size and the degree of coverage of the NP surface have been shown to depend on the surface chemistry of the NPs^5^. PC formation on NPs is basically an universal phenomena for NPs dispersed in biological fluids and it is even observed in NPs with a stealth coating, i.e. modified with molecules like polyethylene glycol or sugars that result in a limited interaction with proteins^6,7^. PC is the ultimate interface with the biological environment and plays a crucial role in cell-NPs interactions. Cell uptake, cytotoxicity and intracellular trafficking have been widely reported to be affected by changes in PC composition or stability^8–11^. In a recent work, we have shown that the intracellular aggregation of NPs correlates to the antifouling character of the coating^12^. Modulating the PC composition through changes in the chemistry of the NP surface seems to be a promising strategy to guide the *in vivo* behavior of NPs as the presence of certain proteins on their surface can direct NPs towards specific intracellular pathways^13,14^.

However, little is known about the intracellular fate of the protein corona around NPs. *S*tudies *in vitro* have shown that the PC is a dynamic entity^11,15,16^. The *hard corona*, the closest layer of proteins to NPs surface, is formed by the proteins that have the highest affinity for the NPs and exchange slowly with the surrounding media. On top of the hard corona, there are proteins weakly associated with the NPs that are easily exchanged; these are named as *soft corona*. Recently, *Bertoli* and coworkers combined flow cytometry and confocal microscopy to study the uptake and trafficking of fluorescently labeled protein corona around NPs in fixed cells^17^. The authors were able to demonstrate that the PC is mainly retained until NPs reach the lysosomes. After that point, degradation occurs and protein fragments either remain in the vesicles or leak in the cytosol.

Fluorescence correlation spectroscopy (FCS) is a spectroscopic technique that provides information on the diffusion of fluorescent molecules and objects in different matrixes, including cells^18,19^. In FCS fluctuations in the intensity of fluorescence coming from molecules or objects in nanomolar concentration range are recorded inside a confocal volume. Diffusion times are derived from the correlation analysis of the fluctuations of fluorescence intensity. Diffusion coefficients can be related to the size or molecular weight of the diffusing species. Additional information on the local micro-environmental conditions, i.e. local viscosity and temperature can be obtained from the diffusion times, as well as the state of aggregation of the diffusing species^20–22^. From FCS data it is also possible to obtain affinity constants among molecules if at least one of them is fluorescent. FCS has been often used to characterize the colloidal stability of NPs in terms of hydrodynamic size in different biological fluids^23–25^ and to describe the structure and composition of PC^26^. However, few works have dealt with the intracellular fate of NPs applying FCS^27^ and none with the intracellular fate of PC.

A variation of the FCS technique is the cross-correlation spectroscopy (FCCS). FCCS can detect dynamic interactions between two molecules or objects labeled with two spectroscopically different dyes, provided that there is no overlapping of their fluorescence spectra^20,28^. FCCS has been widely applied to study protein-protein interactions inside live cells^29–37^and it could provide valuable information on the fate of PC around NPs if both NPs and proteins are properly labelled.

In this work, we apply FCCS for the first time to study the intracellular stability of the PC when it is formed from a protein with a specific affinity for the NP coating. We have studied the interaction of glucosamine modified NPs with Concavalin A. Glucosamide has a well-known affinity for the lectin Concanavalin A (ConA)^38^. We have studied by means of FCCS, both *in vitro* and in the live cell, the strength of the interaction between the glucosamine Au NPs and ConA. We compared it with the affinity of the NPs for bovine serum albumin (BSA), which has nonspecific interactions with glucosamide, besides being the most abundant protein in plasma and it can as well be taken as a model for the plasma itself. The stability of PC after uptake by cells has important consequences in terms of NPs targeting, trafficking, pharmacokinetics and toxicity. The intracellular stability of the PC will influence the aggregation of the NPs and their translocation and localization in the cell interior. These are fundamental issues for targeted drug delivery and for the systematic release of drugs from NPs. The degradation of the PC will result in the NP surface directly exposed to other biomolecules with potential impact on cell metabolism and toxicity^39,40^.

## Results and Discussion

**Figure 1:**
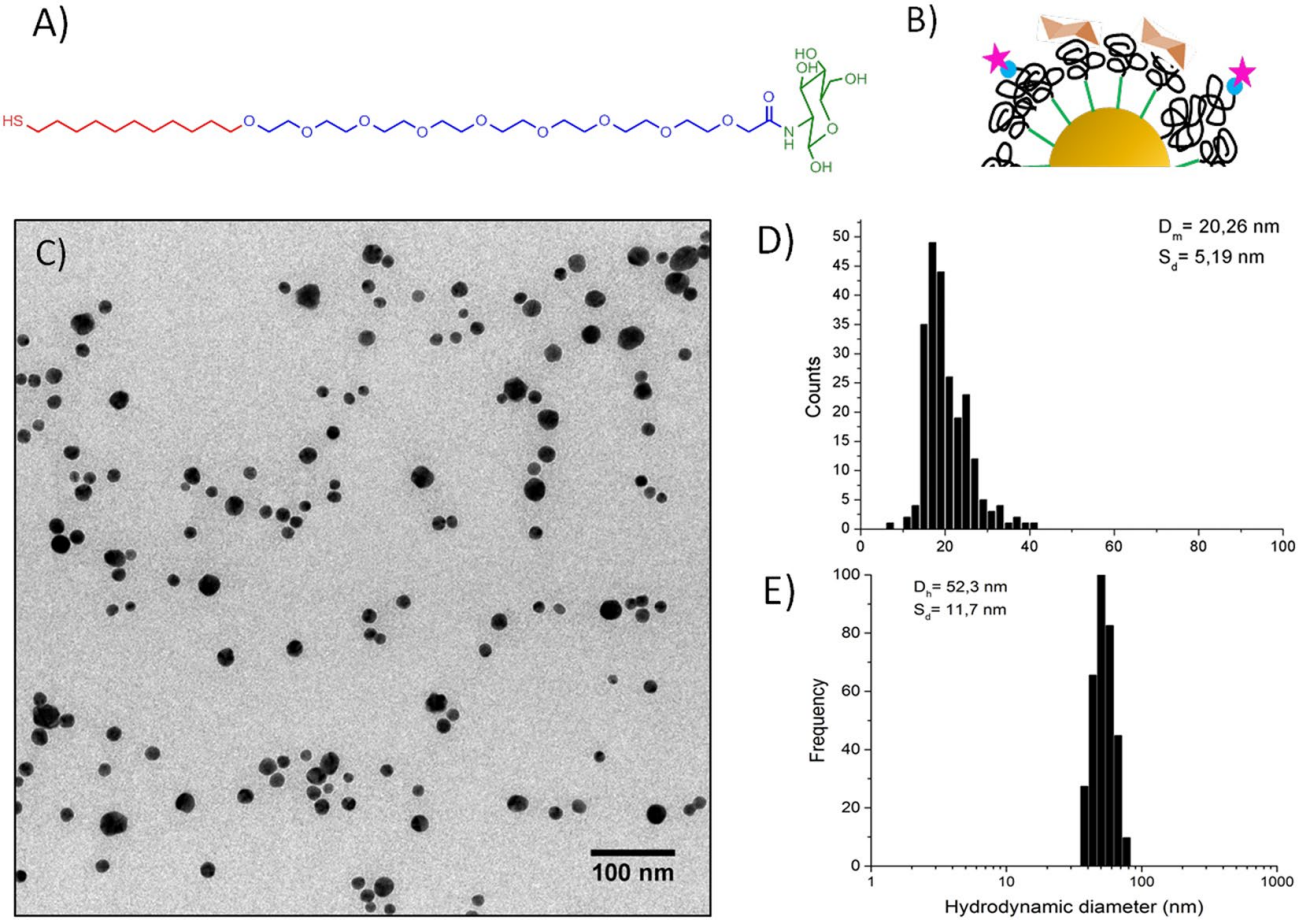
Structure and characterization of glucosamide Au NPs. (**A**) Chemical structure of the glucosamide ligand, highlighting in red the alkyl chain, in blue the poly-ethylene glycol and in green the glucosamide. (**B**) Cartoon of the surface of the Glucosamine Au NPs, the star represents the ATTO-488. (**C**) TEM micrograph of Glucosamine Au NPs and (**D**) size distribution from TEM measured by ImageJ free software. (**E**) DLS hydrodynamic radius distribution by frequency.

We have synthesized gold nanoparticles (Au NPs), approximately 20 nm in diameter, coated with an amphiphilic ligand displaying a hydrophobic alkyl chain and a polyethylene glycol chain (Mw 600 KDa) functionalized with a terminal glucosamide. The ligand was newly synthesized for this work. The simultaneous presence of a short PEG chain and a hydrophobic chain guarantees a tighter ligand packing that impacts the interaction with the others biomolecules as previously shown^41^. We chose to form the PC with only one protein and compare the interaction of the NPs with BSA and ConA. BSA interacts nonspecifically with the NPs and it is used to mimic the interaction of the NPs with serum being the most abundant serum component. ConA, as lectin, binds preferentially glucosamide moieties and we expect a stronger interaction with the NPs coating. The use of a single protein to form the corona is a simplified approach whose validity is confirmed by some recent works. In particular, Tenzer and coworkers^42^ found that the PC changing with time in terms of proteins amount but not composition and Lundquist *et al*.^16^ and Wang *et al*.^43^ reported that a fingerprint of the hard corona is retained when the NPs face several biological environments and during the cellular uptake. In Fig. 1 we report the ligand structure and a sketch of the NP coating (Fig. 1A,B), as well as the size distribution of the NPs as obtained by Transmission Electron Microscopy and Dynamic Light Scattering (Fig. 1C–E, Table S1). NPs and proteins were labelled with ATTO633-NHS and ATTO488-NHS respectively, as described in the SI (Figures S1 and S2). The two dyes have no overlapping emission spectra. ATTO488-NHS was used for labeling both ConA and BSA proteins (Figure S3 in SI).

### *In vitro* fluorescence cross-correlation

**Figure 2:**
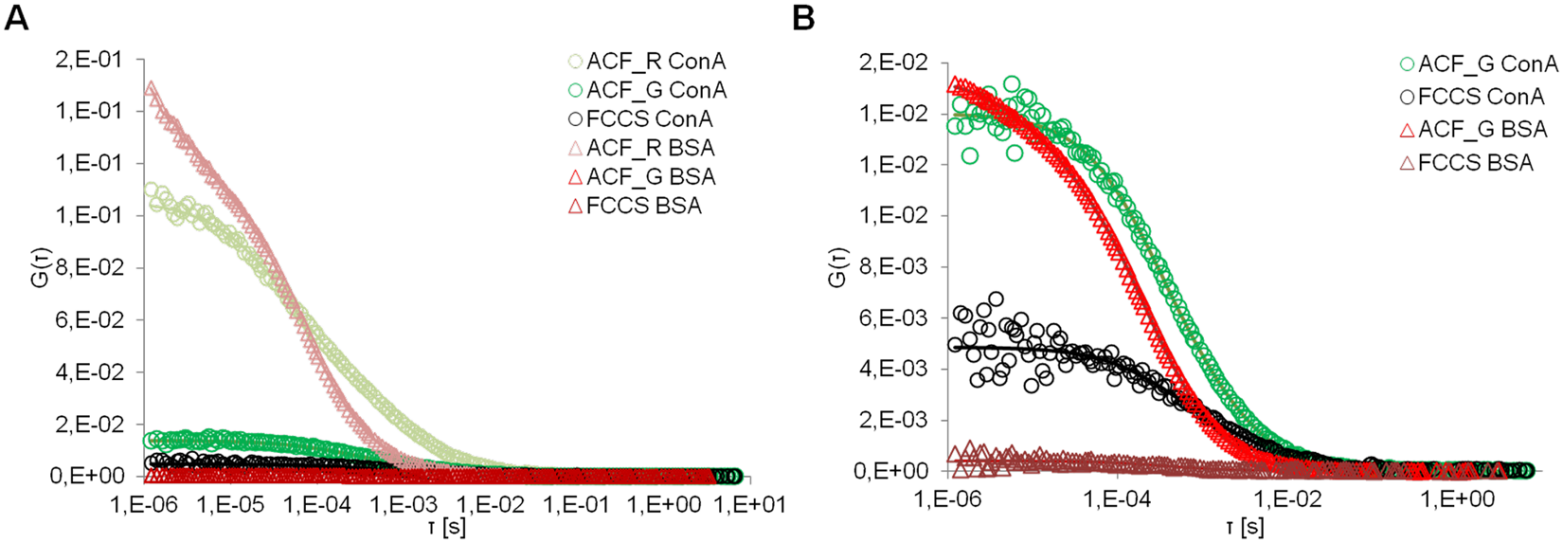
Fluorescence Cross-correlation graphs of glucosamine Au NPs incubated with Concanavalin A (ConA, circle) and Bovine Serum Albumin (BSA, triangle). (**A**) Au NPs were labeled with ATTO633 and detected in the red channel (ACF_R), proteins were labeled with ATTO488 and detected in the green channel (ACF_G). FCCS is the cross-correlation. (**B**) The cross-correlation of BSA and ConA were normalized with respect to the amplitude of the ACF_G. Experiments were performed in triplicates.

We have measured the cross-correlation originated from the direct interaction between ATTO633 labeled NPs and ATTO488 labeled ConA (ATTO488-ConA) or ATTO488 labeled BSA (ATTO488 BSA) (Fig. 2 and Table 1). 0.1 mg/ml of NPs were dispersed in 60 µM protein solutions in Tris-buffered saline (TBS), which contains Mn^2+^ and Ca^2+^, necessary for the glucosamide-Concavalin recognition. Diffusion coefficients are not very different for the two proteins 877 ± 60 and 914 ± 84 µs for BSA and ConA, respectively, and not very different from the diffusion coefficient of the NPs in buffer, 810 ± 214 µs. However, the affinity for ConA is higher because, at the same molar ratio, NPs interacting with ConA display 10fold larger cross-correlation compared to BSA. The cross correlation is normalized respect to the concentration of the proteins, the most abundant specie in solution, to compare the different samples. More details can be found in the FCS and FCCS theory in SI^31^. The hydrodynamic radius reported in literature for BSA in around 3.4–3.5 nm^44^. The hydrodynamic radius of Con A in its dimeric form is 3.3 nm; in the quaternary form is 4.4 nm^45^. The NPs d_H_ does not increase after corona formation as expected. When the proteins are added to the NPs colloidal suspension two effects takes place. On one side, the NPs are coated by the protein corona, increasing the hydrodynamic diameter. Contemporaneously, the interaction between proteins and NPs separates small NPs aggregates present in solution, reducing the effective d_H_. This hypothesis is supported by the fact that the standard deviations are drastically reduced when proteins are added, suggesting that the NPs hydrodynamic dimensions are becoming more homogeneous. The sum of the two effects is still leading to an increase of the d_H_, but smaller than expected. Moreover, the stabilizing ligand has a fundamental importance in the interaction with the proteins. The ligand, employed for this work, is new and the dynamics of its interaction with proteins has not been studied yet in details. Consequently, is not possible for us to foresee if the protein corona will be constituted of a mono or multilayer, or if the proteins could penetrate in the organic coating, as it is happening for longer PEG chains^46^.

**Table 1:** Diffusion times,derived diffusion coefficients and hydrodynamic diameters (D_c_ and d_H_) of the NPs in buffer TBS (Ca^2+^ Mn^2+^) and with proteins BSA and ConA obtained from the fittings in Figure S4 in SI and Fig. 2. Measurements were performed *in situ*. The relative cross-correlation amplitude is reported as FCCS/ACF_G. NPs were labeled with ATTO633 and proteins labeled with ATTO488.

	TBS Ca^2+^Mn^2+^			BSA				Con A			
	t_D_ [µs]^a^	D_c_ [µm^2^/s]^b^	d_H_^c^ [nm]	t_D_ [µs]^a^	D_c_ [µm^2^/s]^b^	d_H_ [nm]	FCCR/ACF_G	t_D_ [µs]^a^	D_c_ [µm^2^/s]^b^	d_H_^c^ [nm]	FCCR/ACF_G
Au NPs	810 ± 216	12.1 ± 3.0	28.2 ± 7.4	877 ± 60	11.1 ± 0.7	30.8 ± 1.9	0.04	914 ± 84	10.7 ± 1.0	31.8 ± 3.0	0.6

a) Values obtained fitting the raw data by Global fitting of 1-componet 3D-Normal Diffusion function. b) The diffusion coefficient was calculated from equation Equation S4 in SI. c) Obtained applying the Stoke-Einstein equation to D_c_.

### Protein exchange experiments

**Figure 3:**
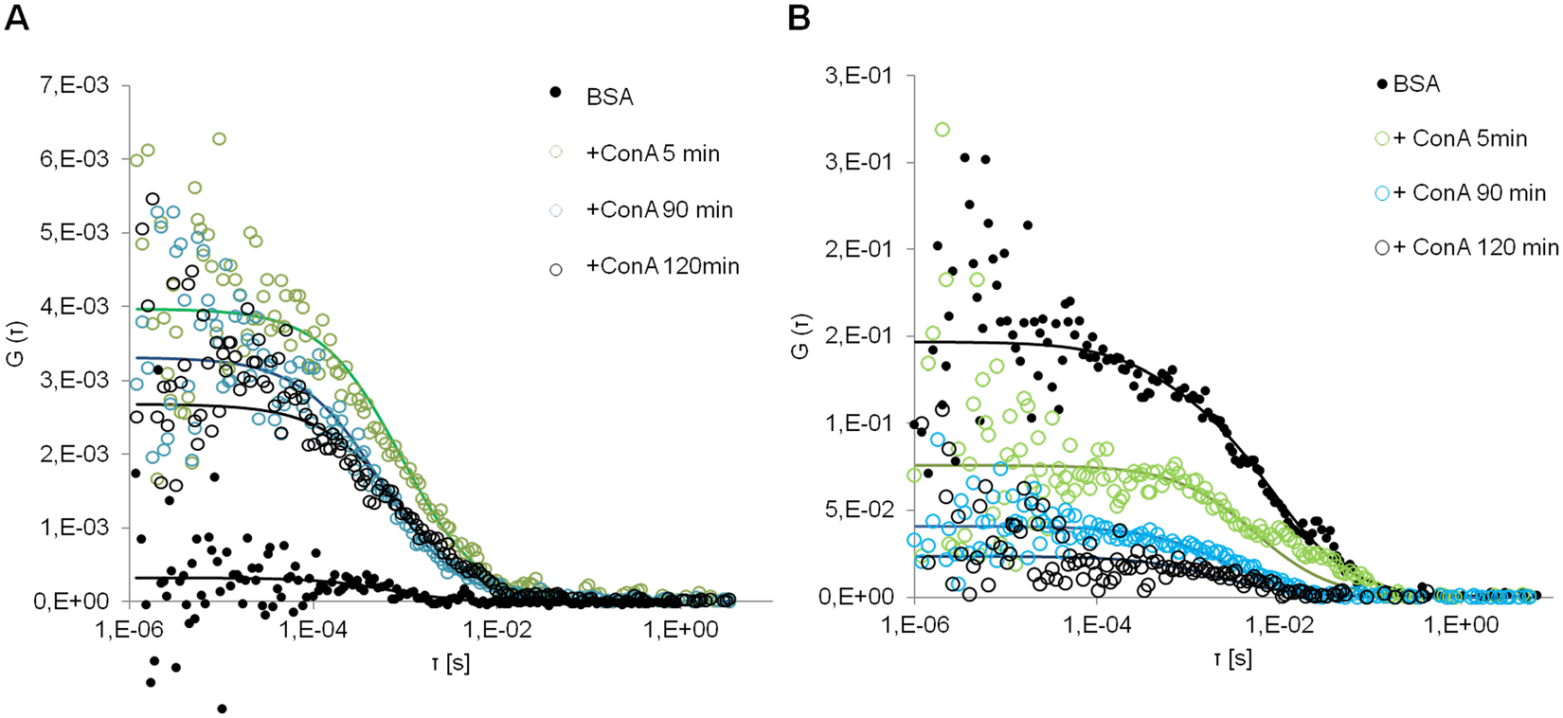
Fluorescence cross-correlation graphs following the protein exchange between ConA and BSA. (**A**)Labeled Au NPs were incubated for one hour with unlabeled BSA, FCCS was measured before and after labeled ConA was added to the dispersion. (**B**) Au NPs were incubated with labeled BSA, FCCS was measured before and after unlabeled ConA was added to the dispersion. The excess of free BSA was removed by centrifugation. The incubation with ConA was followed by FCCS up to two hours. Experiments were performed in triplicates.

The relative affinity of Au NPs for BSA and ConA was evaluated monitoring the exchange of BSA and ConA in the corona with FCCS. In Fig. 3A is reported the cross correlation measured when a PC of unlabeled BSA was formed around the ATTO633 labeled NPs and then the NPs with the BSA PC were exposed to a ATTO488 ConA-solution. The cross correlation varies from a noisy background signal (full black dots) to curves of increasing amplitude. The FCCS amplitude is directly proportional to the concentration of the two of the double-labeled species. An increase of the FCCS curve amplitude indicates an increase in the concentration of double-labeled objects. The relative cross-correlation, defined as the ratio of the cross-correlation to the amplitude of the most abundant species, express the degree of association of the two species in relation to the total concentration of the more abundant specie, see supporting info for theory. In our case, the most abundant species are the proteins, labeled in green. The ratio FCCS/ACF_G, with ACF_G being the autocorrelation function in green channel, gives us the degree of association of proteins to NPs and it can assume a value between 0 and 1, being 1 when all the proteins in the confocal volume are associated to NPs and 0 when there is poor association between the labeled species. Cross-correlation increased when ATTO48- ConA was added to the NPs with preformed corona of unlabeled BSA, resulting in a relative cross-correlation value of 0.53, meaning that a 53% of ConA is bound to the NPs after two hours (Table 2).

**Table 2:** Diffusion times (t_D_), derived diffusion coefficients (D_c_), hydrodynamic diameters (d_H_) and relative cross-correlation FCCS/ACF_G. The diffusion times refer to the curves in Fig. 3. NPs were labeled with ATTO633. Measurements were performed with NPs with PC after the excess of BSA was removed from the dispersion by centrifugation. ConA was added to the PC and measurements were performed *in situ*.

	t_D_ [µs]^a^	D_c_ [µm^2^/s]^b^	d_H_^c^ [nm]	FCCR/ACF_G		t_D_ [µs]^a^	D_c_ [µm^2^/s]^b^	d_H_^c^ [nm]	FCCR/ACF_G
PC BSA	1870 ± 80	5.2 ± 0.2	65.6 ± 2.5	—	PC BSA^d^	1163 ± 230	8.4 ± 1.5	40.6 ± 7.5	0.17
+ConA^d^ 5 min	1242 ± 56	7.9 ± 0.3	43.2 ± 1.6	0.73	+ConA 5 min	2936 ± 180	3.3 ± 0.1	103.4 ± 3.1	0.12
+ConA^d^ 90 min	762 ± 67	12.9 ± 1.0	26.4 ± 2.1	0.63	+ConA 90 min	1162 ± 47	8.4 ± 0.2	40.6 ± 1.0	0.03
+ConA^d^ 120 min	1186 ± 42	8.2 ± 0.4	41.6 ± 2.1	0.53	+ConA 120 min	1287 ± 38	7.6 ± 0.2	45.0 ± 1.2	0.05

(a) Values obtained fitting the raw data by Global fitting of 1-componet 3D-Normal Diffusion function. (b) The diffusion coefficient was calculated from equation Equation S4 in SI. (c) Obtained applying the Stoke-Einstein equation to D_c_. (d) Labeled by ATTO488.

To prove that ConA exchanged with BSA and not only formed additional layers around the PC of BSA, we formed a corona with ATTO488 BSA around the NPs and then, unlabeled ConA was added to the NP dispersion (Fig. 3B). If protein exchange does not take place, the cross-correlation between the ATTO488 BSA and ATTO633 labeled NPs would remain essentially unaltered while the diffusion time would increase. This situation seemed to occur at the beginning of the experiment, after 5 minutes, when the diffusion time of the NPs increased almost three times accompanied by only a slight decrease of FCCS/ACF_G from 0.17 to 0.12. However, after two hours the relative FCCS values decreased to almost zero, from 0.17 to 0.05, and the final diffusion time of the NPs was 1287 ± 38 µs, comparable with the diffusion time observed in the first experiment, 1186 ± 42 µs, after two hours of exposure (see Table 2). We can conclude that ConA substituted almost all the BSA in the corona. The higher values of d_H_, compared to the values reported in Table 1, could be ascribed to the centrifugation process employed to purify the NPs causing a slight aggregation. This phenomenon is reversible as when proteins were added to the dispersion the d_H_ decreased again.

### Fluorescence cross-correlation in cells

**Figure 4:**
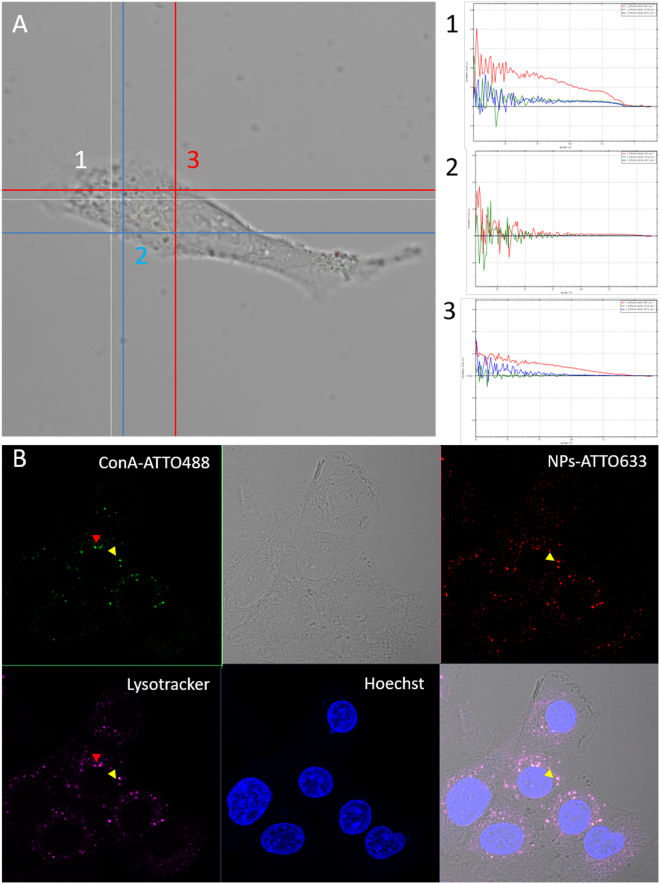
Representative cross-correlation experiment in a live cell. (**A**) The cell was imaged in transmission mode and cross-correlation was measured in distinct locations inside the cell (signed by the numbers 1–2–3). In the example, point 1 corresponded to the cytoplasm and cross-correlation was detected; point 2, on the membrane did not show any fluorescence correlation; point 3, again on the membrane, showed autocorrelation in the red channel (λex = 633 nm, red line) with which is possible to detect NPs labeled with ATTO633, autocorrelation in the green channel (λex = 488 nm, blue line) with which ATTO488 associated to Concanavalin A was detected, but no cross-correlation (green line). (**B**) After cross-correlation measurements, cells are fixed and stained for nuclei (Hoechst, λex = 405 nm) and for acidic vesicles (Lysotracker DND 99-RED, λex = 561 nm). Colocalization of NPs, proteins and vesicles was taking place (yellow arrows) but also partial colocalization between free proteins and vesicles could be observed (red arrows).

**Figure 5:**
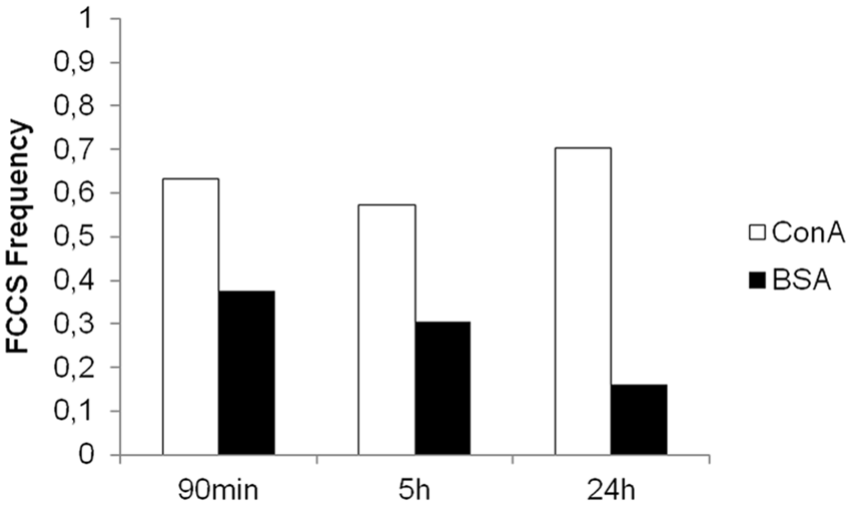
FCCS occurrence at each time-point for measurements performed in cells incubated with NPs with a pre-formed ConA PC and BSA PC and normalized for the total number of measurements. 30, 28, 27 areas were sampled in up to 5 cells exposed to NPs with preformed ConA PC after 90 minutes, and at 5 hours and at 24 hours of incubation, respectively. In the case of NPs associated to BSA, the number of sampled areas was 24, 23, and 31 at 90 minutes, 5 hours and 24 hours of incubation, respectively. Significant differences between ConA PC NPs and BSA PC NPs (Fisher exact test P = 0.0123, P < 0.05).

After verifying that glucosamine Au NPs have a higher affinity for ConA *in vitro*, a cell study was performed. We followed the intracellular fate of the labeled PC either ConA or BSA by means of FCCS. Cell studies were performed with the A549 human adenocarcinoma cell line, which is one of the cell line most frequently used for NP uptake studies. The PC around the gold NPs was formed incubating NPs labeled with ATTO633 in BSA-ATTO488 or ConA-ATTO488 solutions. The excess of proteins was removed by centrifugation and NPs were suspended in serum free media. This was done to avoid that excess proteins from serum would exchange with the proteins in the PC. Cells were grown in full media for 24 hours and then, were incubated for 90 minutes with 75 µg/ml of NPs with a preformed PC in serum free media. Then, the PC evolution was followed intracellularly by mean of FCCS. FCCS measurements were performed in cells just immediately after the incubation was terminated, after 5 hours, and after 24 hours. For each cell studied, measurements were conducted in different cellular regions (Fig. 4A): (1) close to the membrane; (2) in the cytoplasm; (3) in the perinuclear cytoplasm. The perinuclear cytoplasm includes the endoplasmatic reticulum, ER, and late vesicles. The partial colocalization with NPs with preformed PC and lysosomes can be observed in Figs 4B and S5 in SI. Cross-correlation was detected with a significant higher frequency in cells exposed to NPs with a preformed ConA PC, remaining constant at all time-points, i.e. 0.62 at 90 minutes and 0.7 at 24 hours. On the contrary, in cells exposed to NPs with a BSA PC, the frequency of cross-correlation detection decreases with time, from 0.38 after 90 minutes to 0.15 after 24 hours (Fig. 5).

**Figure 6:**
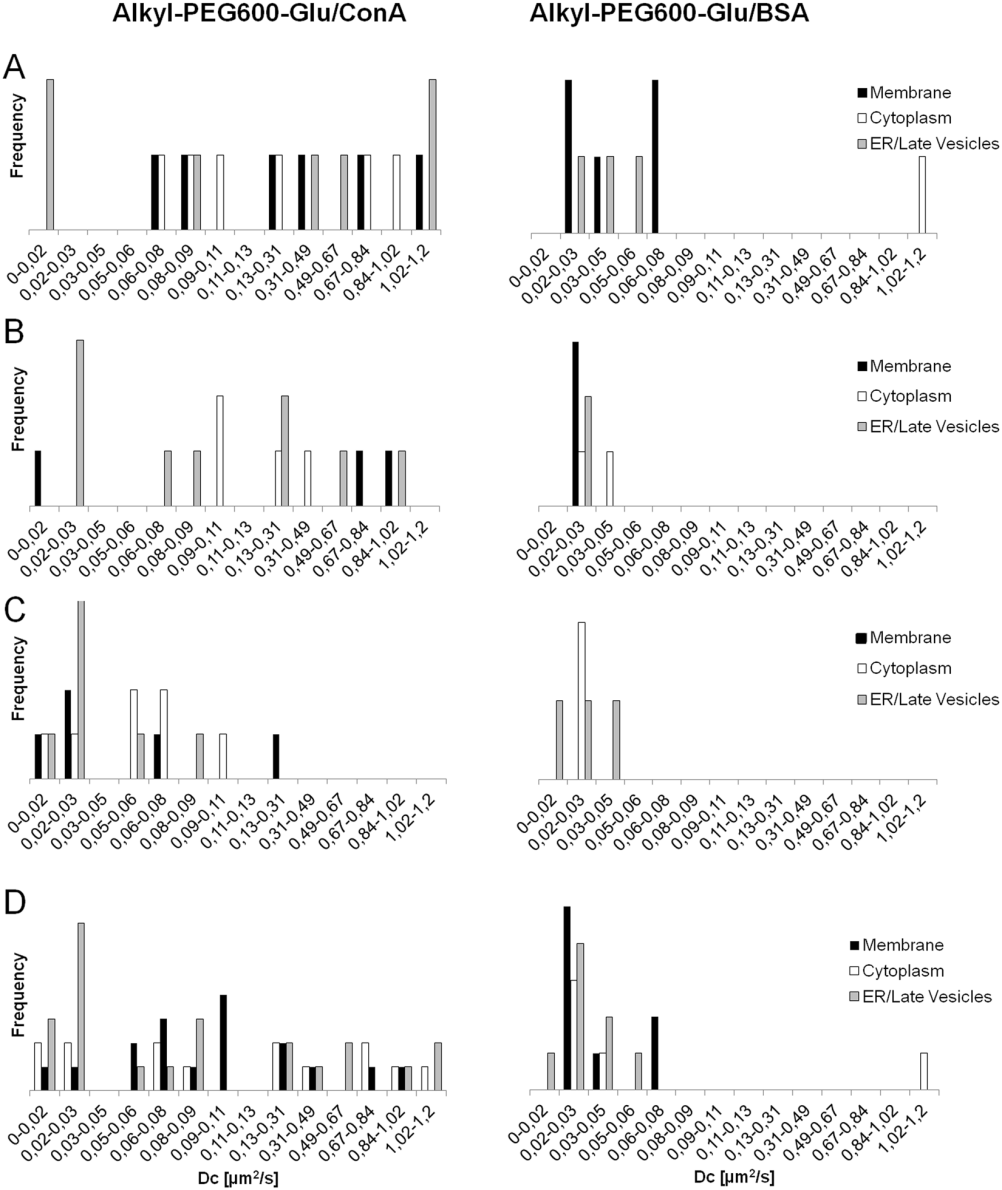
Diffusion coefficients occurrence derived from measurements reported in Fig. 5. Distribution of the diffusion coefficients after 90 min (**A**); 5 hours (**B**) and 24 hours (**C**) of cells growing; (**D**) the diffusion coefficients from all time-points are reported together. Cross-correlation was measured in live cells incubated with Au-Alkyl-PEG600-Glu NPs with preformed ConA PC (left) and BSA PC (right). Measurements were performed in several areas inside the cells: namely membrane (black bar), cytoplasm (white bar), endoplasmatic reticulum (ER) and late vesicles (grey bar).

From the analysis of the cross-correlation curves over 24 hours of cell growth, the diffusion coefficients obtained showed a wide distribution of values ranging from 0.01 to 1.2 µm^2^/s (Fig. 6). The analysis of each time-point is reported. The first time-point presented the widest distribution of diffusion coefficients for NPs with both PCs (Fig. 6A). This is particularly clear for ConA PC. In the case of cells treated with NPs with ConA PC, after 5 and 24 hours of cell monitoring, the diffusion times of the cross-correlating species clearly shifted towards smaller values indicating the formation of slower and/or bigger aggregates. After 5 hours of cell growing, we registered a decrease in cross-correlation detection in correspondence of the membrane from 30% to 17%. After 24 hours of cell growing, all the diffusion coefficients had values below 0.3 µm^2^/s. In cells treated with NPs with BSA PC, the double-labeled species always showed diffusion coefficients in the range 0.05–0.01 µm^2^/s (Fig. 6B,C). After 24 hours FCCS could not be detected in the membrane region for BSA complexes while ConA complexes shifted towards slower diffusion times indicating a different kind of interaction with membrane proteins. BSA-NPs complexes in the cytoplasm evolve with time to diffusing species very slow. At the beginning, they display diffusion coefficients above 1 µm^2^/s, after 5 and 24 hours the diffusion coefficient decreases to 0.02–0.03 µm^2^/s. ConA complexes are continuously detected in the cytoplasm and the evolution toward slow diffusing species progressed over the 24 hours. All these differences highlighted that the two PC NPs followed different uptake and intracellular pathways. Finally, in Fig. 6D the diffusion times for all times are reported together. It is evident that in cells exposed to NPs with ConA PC diffusion coefficients distribute uniformly 0.01 and 1.2 µm^2^/s, while NPs with BSA PC displayed definitely lower diffusion coefficient mainly with values comprised between 0.01 and 0.08 µm^2^/s. ConA complexes were distributed uniformly within all the cell areas sampled. On the contrary, BSA complexes were detected mainly near the membrane and the nucleus, indicating a different transit of the NPs in the cytoplasm, both in terms of location and level of aggregation. The slowest diffusing complexes were detected in the perinuclear area for both NPs.

The use of the Stoke-Einstein Equation for calculating the hydrodynamic radius of the diffusing species does not give reasonable values. In fact, applying a default viscosity of 1.3 cP (average cytoplasm viscosity), sizes in the range 100 nm-50 µm are obtained, which would have sizes comparable with the cells. The NPs in the cell are likely to experience other microenvironments with higher viscosity and/or to be associated to slow/semi-fixed structures, e.g. Golgi apparatus, microtubuli^47,48^. It is worth noticing that in the same area of analysis diffusing species are found that do not show cross-correlation, which can be detected both in the red and green channel (Fig. 4A, point 3). Species with diffusion coefficients larger than 1.2 µm^2^/s are comparable to small NPs aggregates and proteins, from 10 to 40 µm^2^/s, and display a different mobility from the complexes displaying FCCS. The presence of single-labeled diffusing species in intracellular media means that the PC is partially lost through protein exchange and/or degradation.

## Conclusions

In this work, we have been able to follow the intracellular fate of two pre-formed protein coronas around Au NPs functionalized with a thiol-amphiphilic linker and displaying a glucosamide moiety by means of Fluorescence Cross- Correlation Spectroscopy. We demonstrated, first *in vitro*, that there is a higher affinity for ConA than for BSA for the glucosamide Au NPs, and then in the intracellular environment, that the strength of the interaction between NPs and proteins impacts on the evolution of the PC inside the cell. *In vitro*, the dynamic exchange of proteins is reduced when the proteins have specific affinities for the NP surface even if BSA, the nonspecific protein, is present in a large excess in solution^49^.

In the cell environment, the same NPs experience different uptake and trafficking pathways depending if the pre-formed corona is based on BSA or ConA. Cross-correlation from ConA coated NPs can be still detected after 24 hours of cellular processing. For BSA PCs cross-correlation is always lower and decreases with time. Double-labeled complexes with the diffusion time of single NPs with PC were not recorded. On the contrary, the formation of big and slow diffusing complexes takes place. For NPs with preformed ConA PC aggregation increases as the time proceeds. In this case, the distribution of diffusion coefficients is initially broad, then it narrows and shifts towards longer diffusion times. This trend might be explained by a tendency to form aggregates when the PC is lost. BSA corona was lost faster and aggregates of NPs appeared earlier and in larger number in the cells.

Very few studies have been reported on the intracellular dynamics of protein corona. None has reported untill now the direct impact of NP surface affinity for a protein on the trafficking of NPs. The use of cross-correlation spectroscopy allowed us to follow the protein corona evolution inside cell compartments. We could show that the affinity between NP and proteins can assume a significant weight in the intracellular fate of PC and NPs. These findings have important implications in terms of the design of drug delivery vectors addressed to specific cell organelles and to understand toxicological end-points of NPs. The use of a pre-formed corona to reduce the toxicity of the NPs and to address them specifically to cells and organelles has been recently proposed recently^14^. For example, Chen and coworkers studied the role of PC in NPs binding of C3 complement for NPs opsonization^50^. They conclude that, beside the dynamic nature of the PC *in vivo*, controlling its composition in the first place might allow the modulation of complement activation. Our approach for studying the intracellular fate of those complexes could represent an immediate and easy way to screen successful drug delivery vectors. However evidently this approach has limitations since it does not take into account the complexity of the *in vivo* conditions (e.g. blood flow, organs microenvironments)^51,52^.

## Experimental Section

### Nanoparticles synthesis

A water solution of HAuCl_4_•3H_2_O (7.5 ml, 10 mM), sodium citrate (9 ml, 68 mM), and AgNO_3_ (490 μl, 5.9 mM) was prepared and mixed at room temperature for 6 minutes. The pre-incubated mixture was, then, mixed to 250 ml of water at 100 °C. The mixture was stirred at 750 rpm for 1 h. Afterwards, the reaction solution was left to cool at room temperature and 5 ml of glycerol were added. After 10 minutes a second mixture of HAuCl_4_ (7.5 ml, 10 mM), sodium citrate (10 ml, 34 mM) and AgNO_3_ (426 μl, 5.9 mM) was pre-mixed for 6 minutes and then added to the reaction solution, immediately followed by a hydroquinone solution (8 ml, 91 mM). Then, the colloidal solution was left to age for 1 h, stirring at 750 rpm. The obtained Au NPs were directly functionalized without any further concentration or purification. Au- Alkyl-PEG_600_-Glucosamide NPs were obtained by adding (under argon atmosphere) 23.5 mg of Alkyl-PEG_600_-Glucosamide and 6.5 mg of H_2_N-PEG_5000_-SH, dissolved in 5 ml of MilliQ water, to the gold colloidal solution. Details on the synthesis of the ligands^41^ are found in SI. The ligand proportions were calculated to obtain 10% of the NPs surface covered with H_2_N-PEG_5000_-SH, considering a foot print of 1.5 nm^2^ for Alkyl-PEG_600_-Glucosamide and of 5 nm^2^ for H_2_N-PEG_5000_-SH^53–55^. The reaction mixture was allowed to stir for further 48 hours at room temperature. The functionalised Au NPs were purified and concentrated to a final volume of 10 ml using Amicon centrifugal filter units. The purification of the system was completed using dialysis tubes with a cut-off of 10 kDa (48 hours, 6 changes of water).

### Functionalization of NPs and proteins

The buffer employed to perform the fluorescent labeling was obtained mixing 20 parts of a PBS buffer (Phosphate-Buffered Saline, pH 7.4) with 1 part of 0.2 M NaHCO_3_ solution, adjusted to pH 9.0 with 2 M NaOH. The labeling buffer should have a pH of 8.3, optimal for the reaction. 3 mg of Au NPs were dissolved into 2 ml of buffer. 1.5 eq of ATTO633 NHS ester (ATTOTECH-BIO, stocked in 1 mg/ml DMSO Dry solution) was added for each free amino groups present in the reaction mixture. The mixture was sonicated for the first 10 minutes and then let to react for 1 h at R.T. under vigorous stirring. The excess of ATTO633 NHS ester was removed by performing 3 centrifugal filtrations on Amicon centrifugal filters (30 kDa MWCO) and using GE Healthcare PD-10 column. Proteins were labeled using ATTO488 NHS ester (ATTOTECH-BIO) using a 4fold molar excess of dye. The labeling ratio was between 1.5–1.8.

### Characterization

The size of Au NPs was determined by TEM using a JEOL JEM 1400 (120 kV) microscope. The TEM samples were prepared by deposition of the nanoparticle suspension (10 μL) onto a carbon-coated microscopy copper grid. Dynamic Light Scattering (DLS) measurements were performed employing a Malvern Zetasizer Nano ZS90. Specimens were filtered with a cellulose acetate syringe filter (0.22 μm) before to load the cuvette. At least three independent measurements of 10 runs (10 s each one) were performed for each sample. A reduced volume plastic cuvette was employed for DLS experiments loaded with 450 μl of sample. A capillary zeta cell was used for ζ-potential measurements loaded with 1 ml of sample. UV-vis spectroscopy (Spectrophotometer Bio UV-Vis V630 Jasco) was performed using disposable cuvette with 1 cm optical path length. Experiments were performed in triplicate at 25 °C. Fluorescence spectra were registered employing Fluoromiter Fluorolog-TSPC (Horiba-Jovin Ivone). NPs were excited at 635 nm wavelength with a 2 nm slit and 5 mediated accumulations, to enhance the signal to noise ratio. The fluorescence signal was acquired starting from 660 nm. A disposable cuvette with 1 cm optical path length was used for the measurements.

### Protein corona formation for *in vitro* studies

0.1 mg/ml of Au NPs labeled by ATTO633 were dispersed in 60 µM protein solutions in TBS buffer, which contains Mn^2+^ and Ca^2+^. The mixture was kept under agitation 1 hour at 37 °C. Measurements were performed *in situ*.

### Protein corona formation for exchange experiment

0.1 mg/ml of NPs were dispersed in 100 µM BSA solutions in TBS buffer, which contains Mn^2+^ and Ca^2+^. The mixture was kept under agitation 1 hour at 37 °C. The excess of protein was removed centrifuging 10 minutes at 8000×g at 4 °C (Allegra X-22, Beckman Coulter). The PC NPs were suspended in TBS. To test the exchange of BSA with ConA, ConA dispersion in TBS was added to reach a final concentration of 100 µM.

### Protein corona formation for cell studies

Au NPs labeled by ATTO633 were incubated for 1 h at 37 °C with 60 µM ConA and 100 µM BSA, both labeled by ATTO488. The excess of free protein was removed centrifuging 10 minutes at 8000×g at 4 °C (Allegra X-22, Beckman Coulter). The pellet was resuspended at a concentration of 75 µg/ml of NPs in Leibovitz L-15 medium, serum free and phenol red free (ThermoFisher Scientific).

### Cell culture

50.000 A549 cells were seeded on Nunc^TM^ Lab-Tek Chambered Coverglass (purchased from Thermo Fisher Scientific) and grown in a humidified atmosphere at 37 °C with 5% CO_2_ for 24 hours using RPMI full media (ThermoFisher Scientific). Cells were washed by PBS three times and exposed to 75 µg/ml of PC Au NPs obtained as described above. Controls cells were incubated only with the proteins and the NPs. The incubation lasted 90 minutes. After that, cells were washed three times with PBS and were tested for FCCS in Leibovitz L-15 media serum free and phenol free. Cells tested for FCCS at 5 hours and 24 hours were incubated in RPMI full media. Before performing FCCS measures, cells were washed three times with PBS and incubated with Libovitz L-15 media serum free and phenol free. After FCCS measures cells were labeled and fixed for imaging. Lysosomes and acidic vesicles were stained by LysoTracker® Red DND-99 (ThermoFisher Scientific) following the suggested protocol (incubation for 30 minutes at 37 °C with 50 nM solution in full RPMI phenol red free). Cells were washed x3 with PBS and fixed with 3% paraformaldehyde incubating 2 min at 37 °C. Two washes of 5 minutes were performed. Nuclei were stained by Hoechst incubating 5 min at RT. Fixed and stained cells were kept in PBS.

### Confocal microscopy and Fluorescence cross-correlation

Confocal microscopy imaging and FCCS were performed with Confocal Microscope ZeissNLO 880 (Carl Zeiss Gmbh). Acquisition and analysis are controlled by Zen black software. For imaging, the following laser sources were user: the 405 laser, the Argon laser at 488 nm, DPSS at 561 nm and the HeNE laser at 633 nm. The detectors used were GaASP for 405, 488 and 561 and PTM for 633. The 488 channel was coupled with transmission, T-PTM. The objective used was Plan/ Apochromat 63 × /1.4 Oil DIC M27. For FCCS, excitation sources were the Argon laser at 488 nm and the HeNE laser at 633 nm. GaASP and PMTr detectors for single fluorescence molecules detection and dynamic characterization were used. Measurements were performed with a Zeiss C-Apochromat 40_, NA 1.2 water immersion objective. For FCCS in live cell 2 simultaneous fluorescence channel detection coupled with transmission T-PMT were used. NuncTM Fluorescence emission was detected in the range 500–560 nm and 650–710 nm. 300 µl of dispersion were measured in Lab-Tek Q5 Chambered Coverglass (Thermo Fisher Scientific). QuickFit 3.0^56^ free software was used for FCCS data analysis. For further details on FCCS theory, experimental and data analysis refer to SI.

## Electronic supplementary material

Supporting information
