# Supplementary material for: Impact of ConcanavalinA affinity in the intracellular fate of Protein Corona on Glucosamine Au nanoparticles
Source: Sci Rep. 2018 Jun 13;8:9046. doi: 10.1038/s41598-018-27418-w (PMC5998083; doi:10.1038/s41598-018-27418-w)
Supplement: Supplementary file 1 — Supporting information [file 41598_2018_27418_MOESM1_ESM.docx]

Supporting Information

Impact of  ConcanavalinA  affinity in the intracellular fate of Protein Corona on Glucosamine Au nanoparticles

Desirè Di Silvio, Alessandro Silvestri, Luigi Lay, Laura Polito, Sergio Enrique Moya^*^

**Materials and Methods**

HAuCl_4_·3H_2_O, AgNO_3_, hydroquinone, 1,3,4,6-Tetra-O-acetyl-2-amino-2-deoxy-β-D-glucopyranose hydrochloride, 1-Ethyl-3-(3-dimethylaminopropyl)carbodiimide (EDC), N,N'-Dicyclohexylcarbodiimide (DCC), N-Hydroxysuccinimide (NHS), 1,4-Dithiothreitol (DTT), TEA and NaOMe were purchased from Sigma-Aldrich, and used without further purification. HAuCl_4_·3H_2_O was stored at 4°C, shielded from light, as 10mM solution. AgNO_3_ 10 mM and hydroquinone solutions were freshly prepared before every synthesis (avoiding the exposition to the light). HS-PEG_5000_-NH_2_ was purchased from Rapp Polymer GmbHand stored under dry argon atmosphere at -20 °C. HS-Alkyl-PEG_600_-COOH (([1-mercaptoundec-11-yl]PEG_600_)-acetic acid) was synthesized by Chorisis srl. (Varese, Italy) following a literature reported procedure(L. Maus, O. Dick, H. Bading, J. P. Spatz and R. Fiammengo, ACS Nano, 2010, 4, 6617-6628).

**Transmission Electron Microscopy (TEM):** The sizes of AuNPs were determined by TEM using a JEOL JEM 1400 (120 kV) microscope. The TEM samples were prepared by deposition of the nanoparticle suspension (10μL) onto a carbon-coated microscopy copper grid.

**Dynamic Light Scattering (DLS)** measurements were performed employing a Malvern Zetasizer Nano ZS90. Specimens were filtered with a cellulose acetate syringe filter (0.22 μm) before to load the cuvette. Each sample was equilibrated for 2 min prior to measure. At least three independent measurements of 10 runs (10 s each one) were performed for each sample. A reduced volume plastic cuvette was employed for DLS experiments loaded with 450 μl of sample. A capillary zeta cell was used for ζ-potential measurements loaded with 1 ml of sample.

**UV-vis spectroscopy** (Spectrophotometer Bio UV-Vis V630 Jasco) was performed using disposable cuvette with 1 cm optical path length. The experiments were performed in triplicate at 25 °C.

**Fluorescence spectra** were registered employing Fluorometer Fluorolog-TSPC (Horiba-Jovin Ivone). The NPs were excited at 635 nm wavelength with a 2 nm slit and 5 mediated accumulations, to enhance the signal to noise ratio. The fluorescence signal was acquired starting from 660 nm. A disposable cuvette with 1 cm optical path length was used for the measurements.

**Figure S1. Synthesis of ([1-mercaptoundec-11-yl]PEG_600_)-acetic acid glucosamide tetracetate**

The reaction was performed in dry conditions under argon atmosphere. HS-Alkyl-PEG_600_-COOH (152 mg, 0.23 mmol, 1 eq) was dissolved in 1ml of DCM dry. EDC (88 mg, 0.46 mmol, 2 eq) was added, followed by NHS (53 mg, 0.46 mmol, 2eq). The reaction was stirred at room temperature for 30 min, then 1,3,4,6-Tetra-O-acetyl-2-amino-2-deoxy-β-D-glucopyranose hydrochloride (443 mg, 1,15 mmol, 5 eq) dissolved in 1 ml of DCM and 160 μL TEA, were added to the reaction mixture. The reaction mixture was stirred for 1,5 hours at 50 °C and then at room temperature overnight. The reaction was monitored via TLC (AcOEt). The solvent was then evaporated at reduced pressure and the product purified by flash chromatography (DCM/MeOH 9/1), affording 110 mg of product. According to ^1^H-NMR, ~30% of the PEG chains carry the glucosamide moiety.

**^1^H NMR (400 MHz, CDCl_3_)** δ = 7.29 (s, 1H, N***H***), 6.61 (d, J=8Hz, 1H, **H1**), 4.98 (m, 2H), 4.40-4.10 (m, 5H), 3.97 (s, 2H, -O-C**H_2_**-C=O), 3.80-3.55 (m,60H, O-C***H***_2_-C***H***_2_-O), 3.47 (t, J=6.8 Hz, 2H, CH_2_-CH_2_-C***H***_2_-O), 3.38-3.25 (m, 2H, C***H***_2_-NH), 2.50-2.25 (m, 2H, HS-C**H_2_**-), 2.05-2.15 (m, 12H, C**H_3_**), 1.61- 1.58 (m,6H, -C**H2**-alkyl), 1.40-1.20 (m, 16H, -C**H2**- alkyl).

 **Figure S2. Synthesis of ([1-mercaptoundec-11-yl]PEG_600_)-acetic acid glucosamide (HS-Alkyl-PEG_600_-Glucosamide)**

([1-mercaptoundec-11-yl]PEG600)-acetic acid glucosamide tetracetate (65 mg) was dissolved in 3mL of dry MeOH after which a solution of MeONa in dry MeOH (1 M, 1.6 mL) was added dropwise. The mixture stirred for 4 hours at room temperature, monitoring the reaction by TLC (DCM/MeOH 8/2). The reaction mixture was then neutralised with Amberlite IR-120 resin in acidic form, filtered and concentrated to dryness. The residue was directly used without further purification, (43 mg, 0.05 mmol, 1 eq) was dissolved into 5 ml of PBS solution at pH 7.4. DTT (40 mg, 0.26 mmol, 5 eq) was added and the reaction mixture left under stirring at 50 °C for 3 hours, under argon atmosphere. The water was removed at reduced pressure and the product was purified by means of Sephadex G10 and used directly to coat gold nanoparticles.

**Synthesis of Au-** **Alkyl-PEG_600_-Glucosamide NPs**

A water solution of HAuCl_4_•3H_2_O (7.5 ml, 10 mM), sodium citrate (9 ml, 68 mM), and AgNO_3_ (490 μl, 5.9 mM) was prepared and mixed at r.t. for 6 minutes. The pre-incubated mixture was, then, mixed to 250 ml of water at 100 °C. The mixture was stirred at 750 rpm for 1 h. Afterwards the reaction solution was left to cool at r.t. and 5 ml of glycerol were added. After 10 minutes a second mixture of HAuCl_4_ (7.5 ml, 10 mM), sodium citrate (10 ml, 34 mM) and AgNO_3_ (426 μl, 5.9 mM) was pre-mixed for 6 minutes and then added to the reaction solution, immediately followed by a hydroquinone solution (8 ml, 91 mM). Then, the colloidal solution was left to age for 1h, stirring at 750 rpm. The obtained Au NPs were directly functionalized without any further concentration or purification. Au- Alkyl-PEG_600_-Glucosamide NPs were obtained by adding (under argon atmosphere) 23.5 mg of Alkyl-PEG_600_-Glucosamide and 6.5 mg of H_2_N-PEG_5000_-SH, dissolved in 5 ml of MilliQ water, to the gold colloidal solution. The ligand proportions were calculated to obtain 10% of the NPs surface covered with H_2_N-PEG_5000_-SH, considering a foot print of 1.5 nm^2^ for Alkyl-PEG_600_-Glucosamide and of 5 nm^2^ for H_2_N-PEG_5000_-SH. [1-3] The reaction mixture was allowed to stir for further 48 hours at room temperature. The functionalised Au NPs were purified and concentrated to a final volume of 10 ml using Amicon centrifugal filter units. The purification of the system was completed using dialysis tubes with a cut-off of 10 kDa (48 hours, 6 changes of water).

**Au-** **Alkyl-PEG_600_-Glucosamide NPs Characterization**

| ζ-potential | -19,8 ± 4,97 mV |
| --- | --- |
| **Hydrodynamic Diameter** | 52,3 ± 11,7 nm |

**Table S1** ζ-potential and hydrodynamic diameter of Au-Alkyl-PEG600-Glucosamine NPs obtained with dynamic light scattering technique.

**Labeling of the Au NPs with ATTO633 NHS ester**

The buffer employed to perform the fluorescence labeling was obtained mixing 20 parts of a PBS buffer (Phosphate-Buffered Saline, pH 7.4) with 1 part of 0.2 M NaHCO_3_ solution, adjusted to pH 9.0 with 2 M NaOH. The labeling buffer should have a pH of 8.3, optimal for the reaction. 3mg of Au NPs have been dissolved into 2 ml of buffer. 1.5 eq of ATTO633 NHS ester (ATTOTECH-BIO, stocked in 1 mg/ml DMSO Dry solution) was added for each free amino groups present in the reaction mixture. The mixture was sonicated for the first 10 minutes and then let to react for 1 h at R.T. under vigorous stirring. The excess of ATTO633 NHS ester was removed by performing 3 centrifugal filtrations on Amicon centrifugal filters (30 kDa cut-off) and using GE Healthcare PD-10 column. NPs were characterized by TEM, DLS, Uv-Vis and Fluorescence Spectroscopy (**Figure S3-4**).

Figure S3 UV-vis spectra of the dye ATTO633, Au-Alkyl-PEG600-Glu and Au-Alkyl-PEG600-Glu-ATTO633 NPs in MQW.

**Figure S4** Fluorescence spectra of the dye ATTO633 and the functionalized dye Au NPs in MQW. Excitation wavelength 635 nm.

**Cell culture**

50.000 A549 cells were seeded on Nunc^TM^ Lab-Tek Chambered Coverglass (purchased from Thermo Fisher Scientific) and grown in a humidified atmosphere at 37 °C with 5% CO_2_ for 24 hours using RPMI full media (ThermoFisher Scientific). Cells were washed by PBS three times and exposed to 75 ug/ml of Au NPs labeled by ATTO633 and covered by a protein corona of ConA or BSA, both labeled by ATTO488 and disperdes in serum free media . Controls cells were incubated only with the proteins and the NPs. The incubation lasted 90 minutes. After that NPs were washed three times by PBS and were tested for FCCS in Leibovitz **L-15** media serum free and phenol free media (ThermoFisher Scientific). Cells tested for FCCS at 5 hours and 24 hours were incubated in RPMI full media. Before performing FCCS measures, cells were washed three times with PBS and incubated with Libovitz **L-15** media serum free and phenol free media. After FCCS measures cells were dyed and fixed for imaging.

**FCS and FCCS**

FCS and FCCS were performed with a Confocal Microscope ZeissNLO 880 (Carl Zeiss Gmbh). Acquisition and analysis are controlled by Zen black software. Excitation sources were the Argon laser at 488 nm and the HeNE laser at 633 nm. GaASP and PMTr detectors for single fluorescence molecules detection and dynamic characterization were used. The microscope objective used was Zeiss C-Apochromat 40_, NA 1.2 water immersion objective. For FCCS in live cell 2 simultaneous fluorescence channel detection coupled with transmission T-PMT were used. NuncTM Fluorescence emission was detected in the range 500-560 nm and 650-710 nm. Cross-talk between the emission spectrum of ATTO488 and the absorption spectrum of ATTO633 was minimized choosing narrow detection ranges as shown in Figure S3 and confirmed <4% according to *Bacia et al. 2007* using Rhodamine123 20nM in MQW (Sigma). FCS and FCCS measures were performed on dispersion of NPs and proteins and in live cells. Lab-Tek Q5 Chambered Coverglass (purchased from Thermo Fisher Scientific) were used.


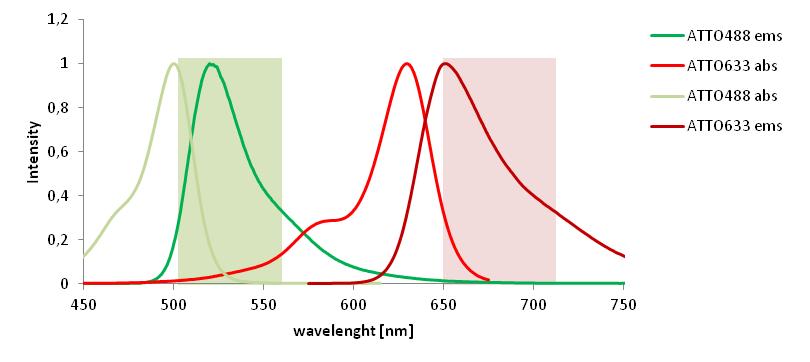


Figure S5 Absorption and Emission spectra for ATTO488 and ATTO633 obtained. The detection ranges fixed in FCCS configuration experiment are highlighted as green area (500-560 nm) and as red area (650-710 nm) assuring a cross-talk <4%.

**Confocal microscopy**

Confocal microscopy imaging was performed with Confocal Microscope ZeissNLO 880 (Carl Zeiss Gmbh). Excitation sources were laser 405, Argon laser at 488 nm, DPSS at 561nm and the HeNE laser at 633 nm. The detectors used were GaASP for 405, 488 and 561 and PTM for 633. 488 channel was coupled with transmission, T-PTM. The objective used was Plan/ Apochromat 63x/1.4 Oil DIC M27.

Measures were performed on dyed fixed cells. Lysosomes and acidic vesicles were stained by LysoTracker® Red DND-99 (ThermoFisher Scientific) following the suggested protocol (incubation for 30 minutes at 37°C with 50nM solution in full RPMI phenol red free). Cells were washed x3 with PBS and fixed with 3% paraformaldehyde incubating 2 min at 37°C. Two washes of 5 minutes were performed. Nuclei were stained by Hoechst incubating 5 min at RT. Fixed and stained cells were kept in PBS.

**FCS and FCCS Theory**

A detailed description of FCS and FCCS can be found in the following articles and reviews: Elson and Magde, 1974; Thompson,1991; Rigler et al., 1993; Elson and Rigler, 2001.

In FCS temporal fluctuations in the fluorescence signals F_i_(t) of emitting species are analyzed using the following correlation function (**Equation S1**):

$$G_{ij}^{F}(\tau)=\frac{<F_{i}(t)\cdot F_{j}(t+\tau)>}{<F_{i}\cdot F_{j}>}$$

or

$$G_{ij}^{\vartheta F}(\tau)=\frac{<{\vartheta F}_{i}(t)\cdot\vartheta F_{j}(t+\tau)>}{<F_{i}\cdot F_{j}>}$$

Where < > denotes the time average, *δF(t) = F(t) - ‹F(t)›* is the fluctuations of *F(t),* *i* = *j* in the case of autocorrelation, and *F*(*t*) is assumed to be constant over long-time averages.

In the case of fluctuations arising from free Brownian diffusion of *i* different species of fluorescent molecules, the autocorrelation functions *G(τ)* was fitted by a normal 3D diffusion model that can be denoted as (**Equation S2**):

$$G\left( \tau\right)=G_{\infty}+\frac{1}{N}X_{back}\cdot\left( \frac{1-\theta_{non}+\theta_{non}e^{\frac{-\tau}{\tau_{non}}}-\theta_{trip}+\theta_{trip}e^{\frac{-\tau}{\tau_{trip}}}}{1-\theta_{non}-\theta_{trip}} \right)\cdot\left[ \left( 1-\rho_{2}-\rho_{3} \right)\cdot g_{1}\left( \tau\right)+\rho_{2}\cdot g_{2}\left( \tau\right)+\rho_{3}\cdot g_{3}\left( \tau\right) \right]$$

Where the factor g_1_(τ) is correspondent to (**Equation S3**):

$$g_{i}\left( \tau\right)=\left( 1+\frac{\tau}{\tau_{D,i}} \right)^{-1}\cdot\left( 1+\frac{\tau}{{\gamma^{2}\tau}_{D,i}} \right)^{\frac{-1}{2}}$$

The background correction is calculated like:

$$X_{back}=\frac{{(I-B)}^{2}}{I^{2}}$$

*I* is the intensity of the signal and *B* is background intensity.

The parameters involved in the function are:

**- G∞:** offset of the correlation function

**- N:** overall particle number (including currently dark particles, for example in triplet state)

**- Θ_trip_, _θnon_:** fractions of the particles in one of the first two non-fluorescent states

**- τ_trip_, τ_non_:** decay times of the first two non-fluorescent states

**- ρ_1_,ρ_2_,ρ_3_ (ρ_1_=1-ρ_2_-ρ_3_):** fractions of the three diffusing components

**- τ_Di_:** diffusion decay time of the i^th^ diffusing component

This model also calculates the diffusion coefficient, if the width *ω_xy_* of the laser focus (xy plane) is known, as (**Equation S4**):

$$D=\frac{\omega_{xy}^{2}}{4\tau_{D}}$$

Also the effective focal volume *V_eff_* is calculated (**Equation S5**):

$$V_{eff}=\pi^{3/2}\cdot\gamma\cdot\omega_{xy}^{3}$$

Given this focal volume, the particle concentration in the sample (**Equation S6**):

$$C=\frac{N}{V_{eff}}$$

From the diffusion coefficient is possible to calculate the hydrodynamic radius of the species applying the Stoke-Einstein Equation (**Equation S7**):

$$D=\frac{K_{B}T}{6\pi\eta r}$$

Where *K****_B_*** is the Boltzmann’s constant, *T* is the absolute temperature, *η* is the dynamic viscosity and *r* the hydrodynamic radius.

In dual-color cross*-*correlation (Schwille et al., 1997) the fluorescence emission signals from the two dyes are separately detected and, in addition to the autocorrelation, the cross-correlation function is calculated (Eq. 1 with *i ≠* *j*). The normalized cross-correlation function is defined as (**Equation S8**):

$$G_{RG}(\tau)=\frac{<F_{G}(t)\cdot F_{R}(t+\tau)>}{<F_{G}(t)\cdot F_{R}(t)>}$$

Where the indexes R and G refer to the species emitting in the red and green channels respectively. In the absence of spectral bleed-through, the cross-correlation function is zero for non-interacting particles. The cross-correlation amplitude is directly proportional to the concentration of the double labeled specie (**Equation S9**):

$$\frac{G_{RG}(0)}{G_{R}(0)\cdot G_{G}(0)}=\rho_{RG}\cdot N_{tot eff}=N_{RG eff}=<c_{RG}>\cdot V_{eff}$$

To facilitate comparisons of cross-correlation curves absolute concentrations of dual-color molecules were normalized by the concentration of the less abundant chromophore species. The ratio of the corresponding cross-correlation amplitudes was termed ‘‘relative cross correlation’’, CC_rel_j_ (**Equation S10**):

$${CC}_{rel\_j}=\frac{{<C}_{ij}>}{<C_{j}>}=\frac{G_{ij}(0)}{{V_{eff}G}_{Ri}(0)\cdot G_{j}(0)}\cdot V_{eff}\cdot G_{i}\left( 0 \right)=\frac{G_{ij}\left( 0 \right)}{G_{i}\left( 0 \right)}$$

For *i, j* = *R, G* (*i≠j*)

CCrel_j is calculated simply by normalizing the cross-correlation amplitude by the lower

of both autocorrelation amplitudes, *G_i,j_(0).*

We used the software QuickFit 3.0^[[1]](#footnote-1)^.

We calculated *ω_xy_* by performing a calibration with the dye of known diffusion time Rhodamine 123 (diffusion coefficient of 440±30 μm^2^/s).

The cross-correlation data were analyzed in Global FCCS Fit Mode applying a 3D Normal Diffusion equation with 1 no-fluorescent state and 1 or 2 diffusing components according to the quality of the fit obtained. The cross-correlation function was fit with only one diffusing component.

Each selected area in the cell was measured with 20 runs of 10 second each. Each track was fit individually and the diffusion coefficients calculated according **Equation S4**.

**Au NPs-ATTO633 in TBS buffer**


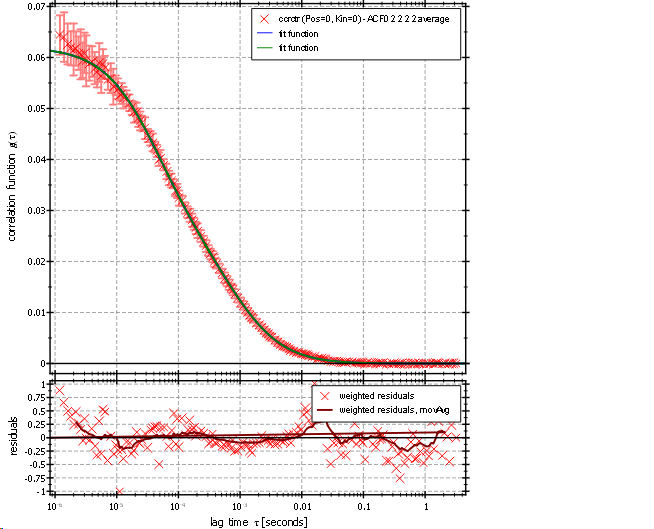


Figure S6 Fluorescence correlation spectroscopy of Gold NPs-ATTO633 in TBS buffer and relative fit with residuals distribution. Chi square of 0.0003.


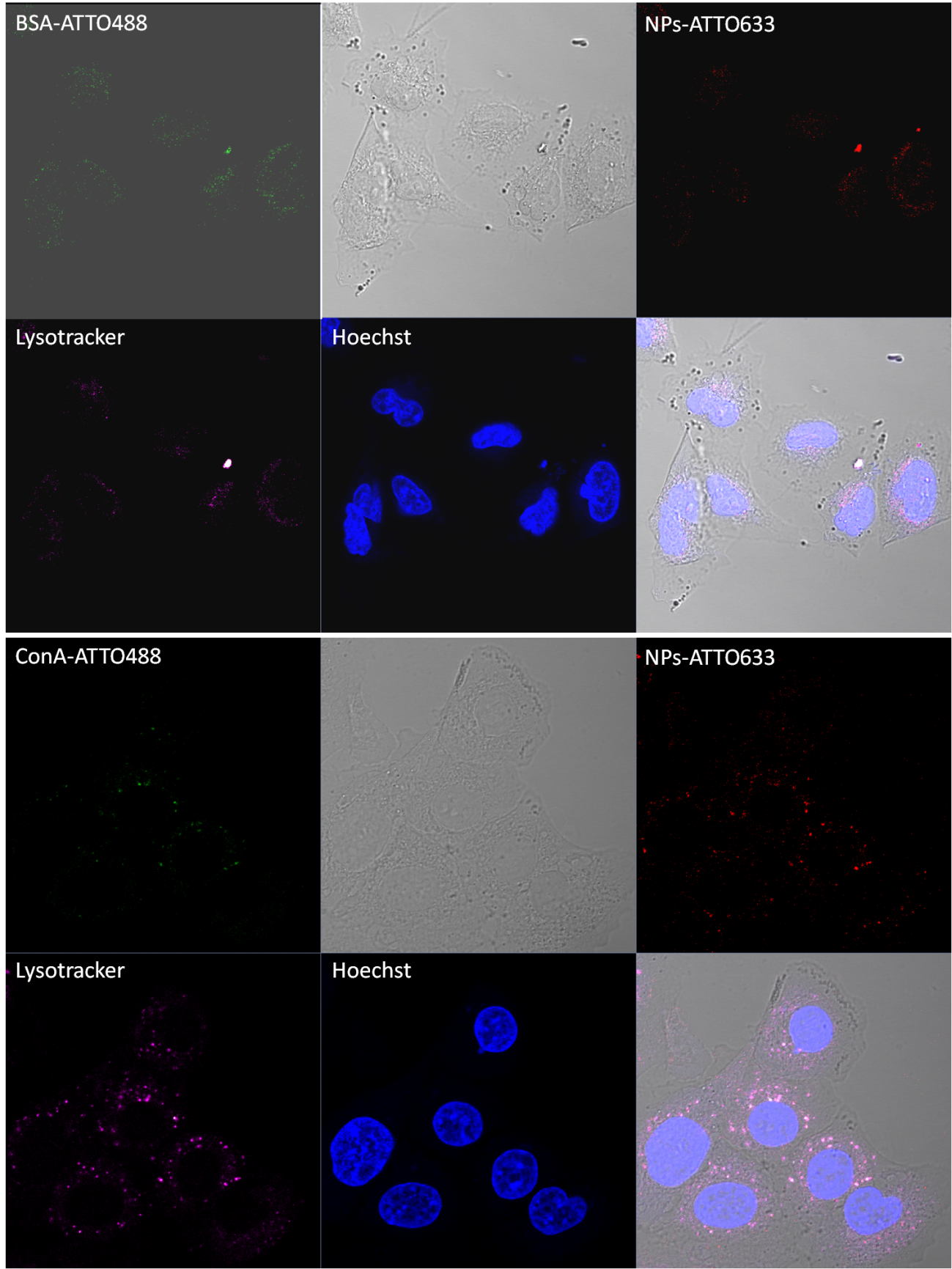


Figure S7 Confocal micrographs of cells incubated with Au-Alkyl-PEG600-GLU associated to BSA and ConA for 90 minutes. From the top, clockwise: Green channel: BSA/ConA-ATTO488; Transmission channel; Red channel: NPs-ATTO633; Combined channel; Blue channel: nucleus-Hoechst; Pink channel: Lysotracker Red DND-99. Zeiss LSM 880 equipped with 63× oil objective lens (1.4 NA).


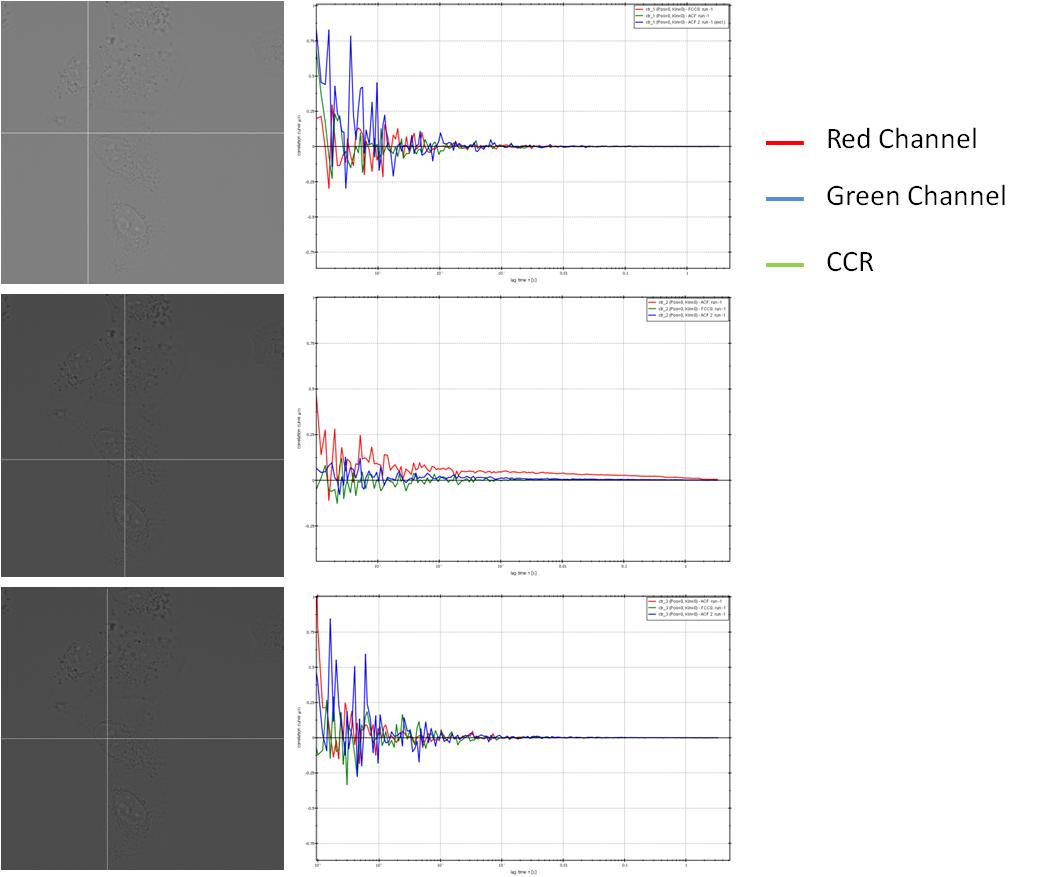


**Figure S8** Representative cross-correlation experiment on a live cell incubated only with serum free media. The cell was imaged in transmission mode and cross-correlation was measured in distinct locations inside the cell.


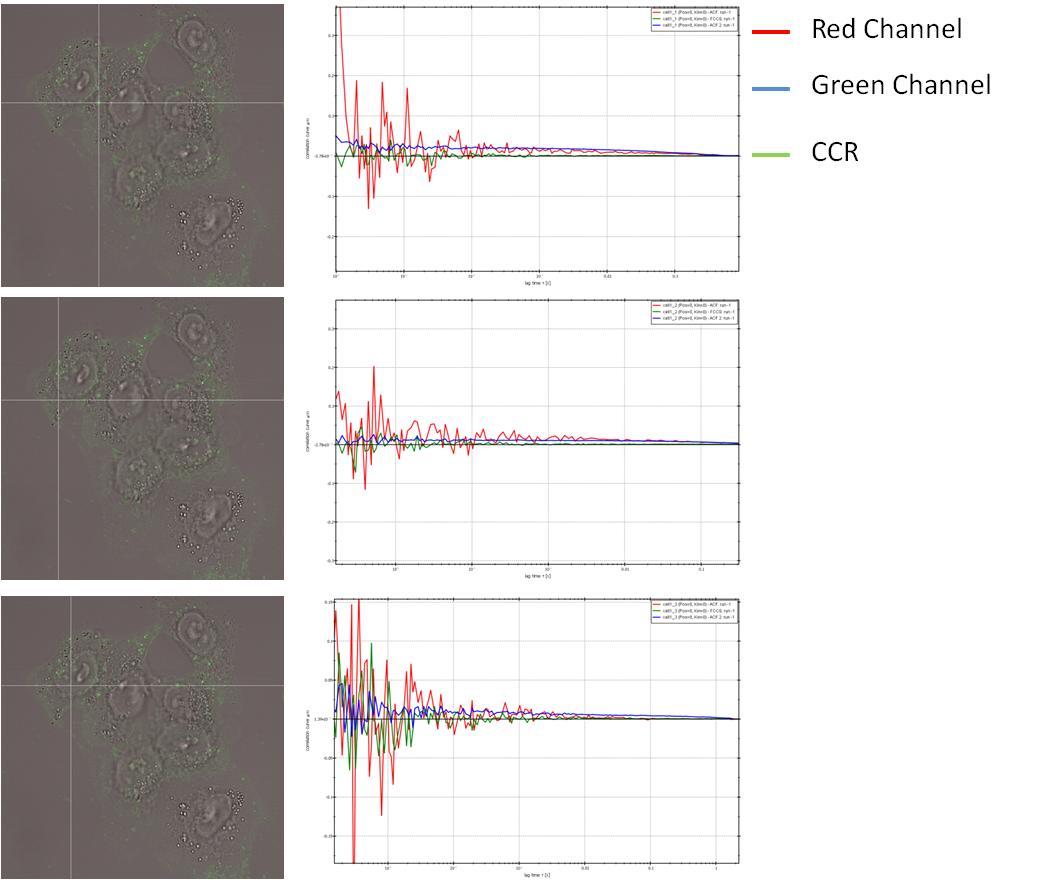


**Figure S9** Representative cross-correlation experiment on a live cell of ConA-ATTO488 alone. The cell was imaged in transmission mode and cross-correlation was measured in distinct locations inside the cell.


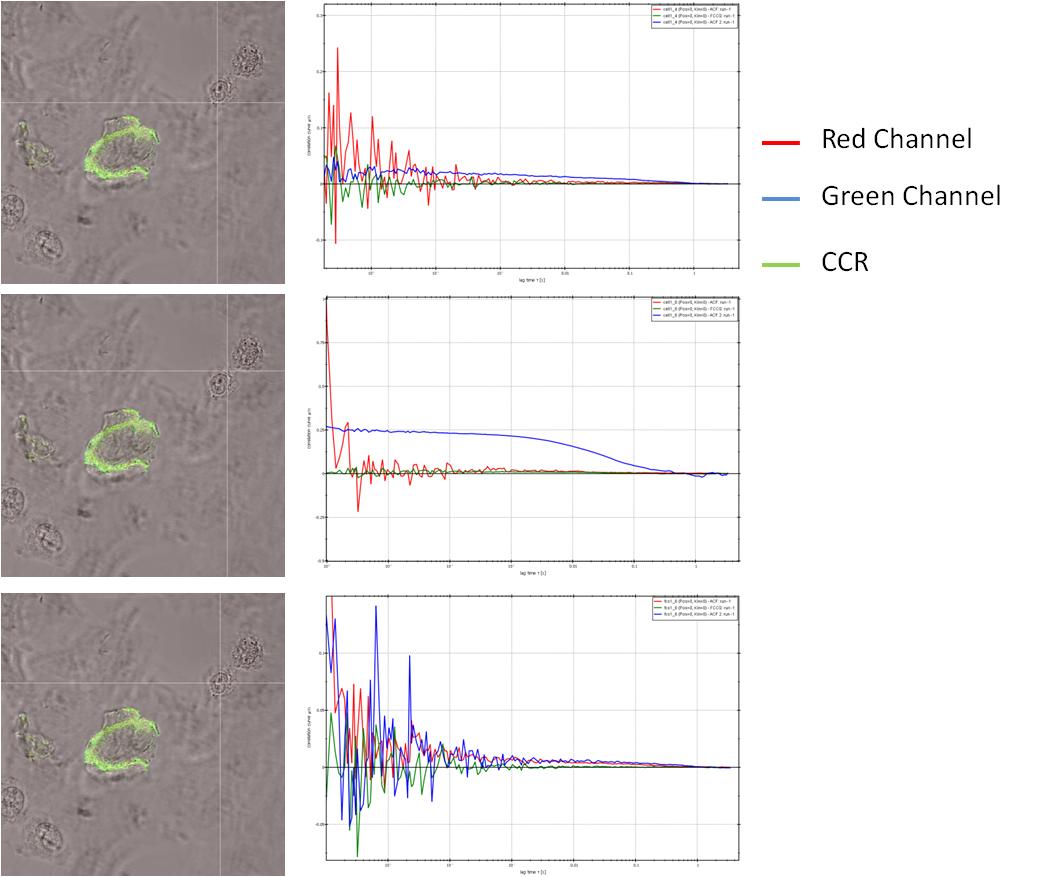


**Figure S10** Representative cross-correlation experiment on a live cell of BSA-ATTO488 alone. The cell was imaged in transmission mode and cross-correlation was measured in distinct locations inside the cell.


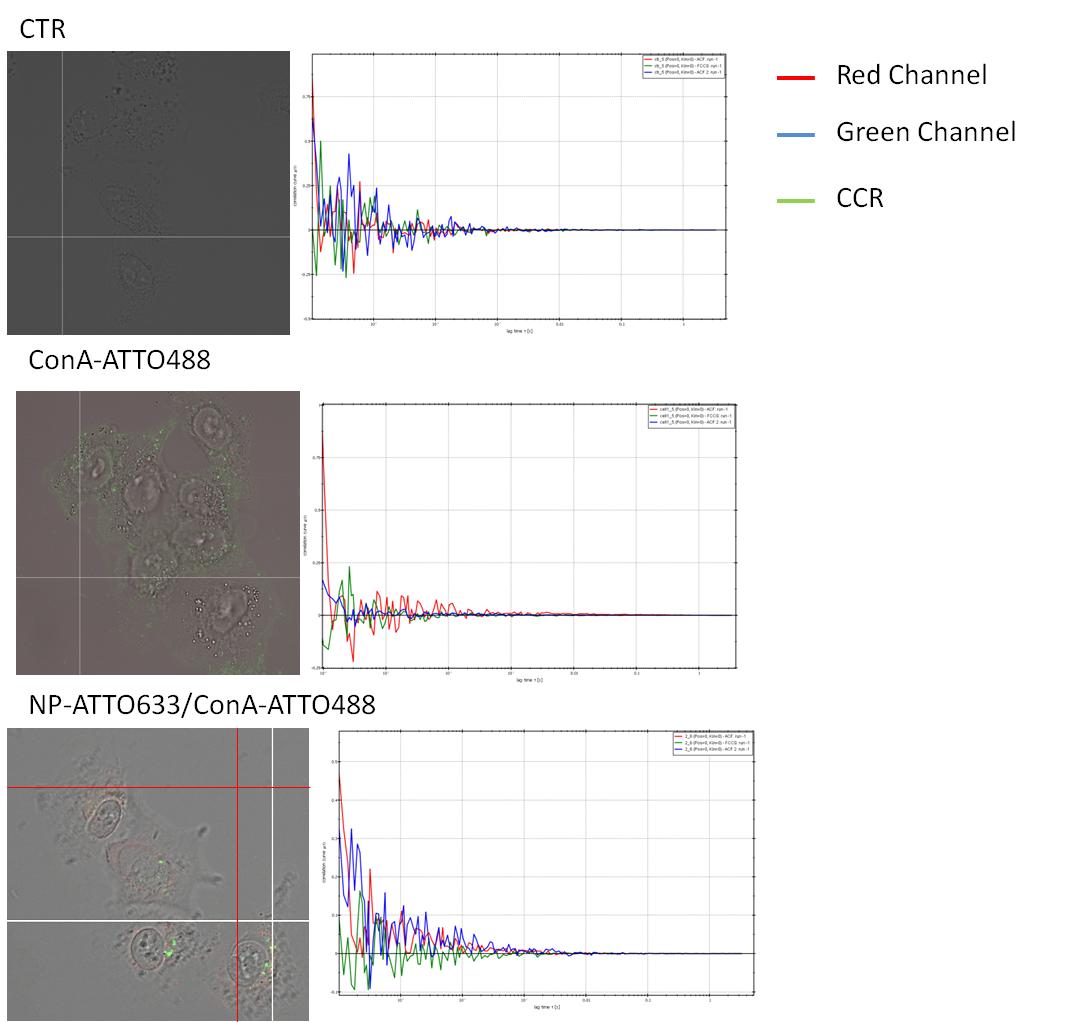


**Figure S11** Representative cross-correlation experiment recorded outside the cells when incubated with serum free media (CTR), with ConA-ATTO488 and with the PC NPs.

1. Jin, J.; Han, Y.; Zhang, C.; Liu, J.; Jiang, W.; Yin, J.; Liang, H., *Colloids and Surfaces B: Biointerfaces* **2015,** *136*, 838-844. DOI http://doi.org/10.1016/j.colsurfb.2015.10.025.

2. Levin, C. S.; Bishnoi, S. W.; Grady, N. K.; Halas, N. J., *Analytical chemistry* **2006,** *78* (10), 3277-3281. DOI 10.1021/ac060041z.

3. Duncanson, W. J.; Figa, M. A.; Hallock, K.; Zalipsky, S.; Hamilton, J. A.; Wong, J. Y., *Biomaterials* **2007,** *28* (33), 4991-4999. DOI 10.1016/j.biomaterials.2007.05.044.

1. Jan Wolfgang Krieger, Jörg Langowski (2010-2017): **QuickFit 3.0 (compiled: 2015-10-29, SVN: 4465): A data** [**evaluation**](tooltip:evaluation) **application for biophysics**, *[web page]* [*http://www.dkfz.de/Macromol/quickfit/*](http://www.dkfz.de/Macromol/quickfit/) *[Accessed on 2017/10/2* [↑](#footnote-ref-1)
